# A96 WHICH IS THE BEST ENDOSCOPIC HEMOSTATIC THERAPY FOR DIEULAFOY’S LESIONS? A SYSTEMATIC REVIEW AND META-ANALYSIS

**DOI:** 10.1093/jcag/gwae059.096

**Published:** 2025-02-10

**Authors:** S Samnani, Y Yuan, G Leontiadis, A Barkun, L Laine, F Tse

**Affiliations:** McMaster University, Hamilton, ON, Canada; McMaster University, Hamilton, ON, Canada; McMaster University, Hamilton, ON, Canada; McGill University, Montreal, QC, Canada; Yale University School of Medicine, New Haven, CT; McMaster University, Hamilton, ON, Canada

## Abstract

**Background:**

Dieulafoy’s lesion (DL) is an uncommon but serious cause of significant gastrointestinal bleeding, typically treated with various endoscopic therapies.

**Aims:**

This systematic review and meta-analysis is the first to evaluate the effectiveness and safety of endoscopic hemostatic therapies for treating DL, including endoscopic band ligation (EBL), endoscopic hemoclip placement (EHP), over-the-scope clips (OTSC), thermocoagulation, topical hemostatic agents (THA) and injection methods (epinephrine, sclerosant).

**Methods:**

We searched MEDLINE, EMBASE, and Cochrane Central Register of Controlled Trials up to March 2024, including randomized controlled trials (RCTs) and observational cohort studies comparing different endoscopic techniques for DL. Two authors conducted study selection, data extraction and quality assessment independently. The primary outcome was 7-day further bleeding. Further bleeding is a composite outcome of failure to achieve immediate hemostasis and rebleeding. Secondary outcomes included failure to achieve immediate hemostasis, rebleeding, overall mortality, adverse events, and additional hemostatic therapy. Pooled risk ratios (RR) with 95% confidence intervals (CI) were calculated using RevMan 5.4. Heterogeneity was assessed by Chi^2^ (P<0.15) and I^2^ tests (>25%). The GRADE approach was used to assess the certainty of evidence (CoE).

**Results:**

We included 7 RCTs, 4 comparative cohort studies, and 27 single-arm cohort studies from 5501 citations. Meta-analyses of EBL compared to EHP showed RRs for 7-day further bleeding in RCTs and cohort studies of 0.66, 95%CI 0.09-5.03 and RR 0.22, 95% CI0.05-0.95, respectively (very low CoE). Additionally, RR for EBL compared to thermocoagulation for 7-day further bleeding in a cohort study was 0.15, 95%CI 0.01- 2.71 (very low CoE). RRs for epinephrine injection alone compared to EBL/EHP and thermocoagulation were 4.37, 95%CI 1.43-13.33 and 10.08, 95%CI 0.63-162.0, respectively (very low CoE). Table 1 shows the results of the standard meta-analyses and proportional meta-analyses.

**Conclusions:**

Epinephrine should not be used as monotherapy for the treatment of DL. Mechanical endoscopic therapies, especially EBL, appear to be an effective option for DL. Further research is needed to assess the roles of OTSC and THA in managing DL and obtain more precise estimates of the effectiveness of EBL, EHP, and thermocoagulation.

**Table: Outcomes in patients with DL treated with different endoscopic interventions.**

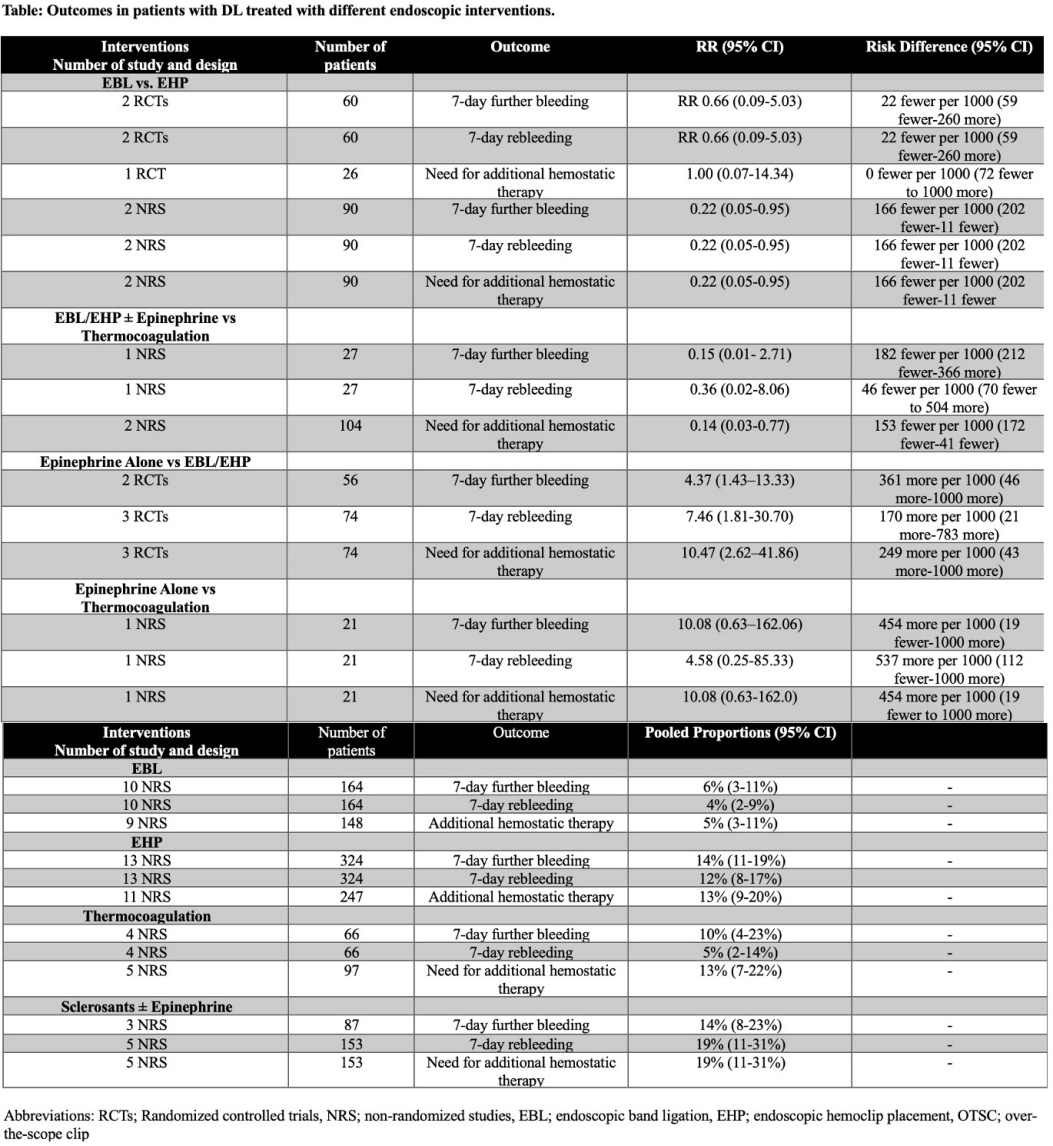

**Funding Agencies:**

**None**

